# Identification of Goldenseal (*Hydrastis canadensis* L.) Habitat and Indicators in Pennsylvania, USA: The Influence of Climate and Site on In Situ Conservation of an Edge of Range Plant Species

**DOI:** 10.1002/ece3.71050

**Published:** 2025-02-28

**Authors:** Ezra Houston, Eric P. Burkhart, Grady Zuiderveen, Xin Chen

**Affiliations:** ^1^ Department of Ecosystem Science and Management The Pennsylvania State University University Park Pennsylvania USA; ^2^ USDA Forest Service, Ecosystem Management Coordination Grand Rapids Michigan USA; ^3^ Appalachian Laboratory Center for Environmental Science, University of Maryland Frostburg Maryland USA

**Keywords:** Forest community types, habitat suitability, in situ conservation, indicator species, Maxent, species distribution modeling

## Abstract

Goldenseal ( *Hydrastis canadensis* L.) is a perennial herbaceous plant native to eastern North America. Commercial harvesting for the medicinal plant trade and habitat loss have led to international conservation concerns. This study sought to understand habitat predilections for the purpose of guiding in situ conservation efforts in Pennsylvania, within the natural range of the species in the northeastern United States. The state's variation in geology and biogeographic location provides an opportunity to examine the influences of edaphic, topographic, and climatic factors on goldenseal habitat suitability here. Maximum Entropy modeling (Maxent) using known occurrence points (*n* = 51) was combined with field plot data (*n* = 28) to identify potential factors associated with goldenseal's distribution in Pennsylvania and to identify vegetative indicators of supportive habitat. Bedrock type and winter temperature were the best predictors of habitat suitability. Suitable bedrock types were base‐rich; a trait confirmed in the field by soil test results showing high calcium and pH levels. However, the influence of bedrock is complicated by overlapping land use legacy. Suitability increased with average winter temperature, peaking toward the upper end of average winter temperatures in Pennsylvania. Community analysis identified 159 woody and herbaceous associates, including indicators of the following supportive rich mesic forest types: “Tuliptree‐Beech‐Maple,” “Red Oak‐Mixed hardwood,” and “Central Appalachian Rich Cove”. Model and field results can be used in tandem to assess site suitability, which was highest on forestlands possessing slightly acidic to neutral loamy soils underlain by base‐rich bedrock types on moist, lower slope positions. Vegetative “indicator” species of these rich‐mesic forests, including *Liriodendron tulipifera* , *Acer saccharum* , *Lindera benzoin* , *Arisaema triphyllum* , and *Botrypus virginianus*, are potentially useful field indicators of supportive habitat for in situ conservation efforts.

## Graphical abstract

Goldenseal ( *Hydrastis canadensis* L.) is a perennial herbaceous plant native to eastern North America. GIS‐based Maximum Entropy (Maxent) modeling using known occurrence points was combined with field plot data to identify potential factors governing goldenseal's distribution in Pennsylvania and identify vegetative indicators of supportive habitat. Model and field results can be used in tandem to assess site suitability.

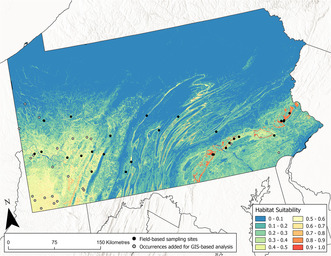

## 1 Introduction

**FIGURE 1:**
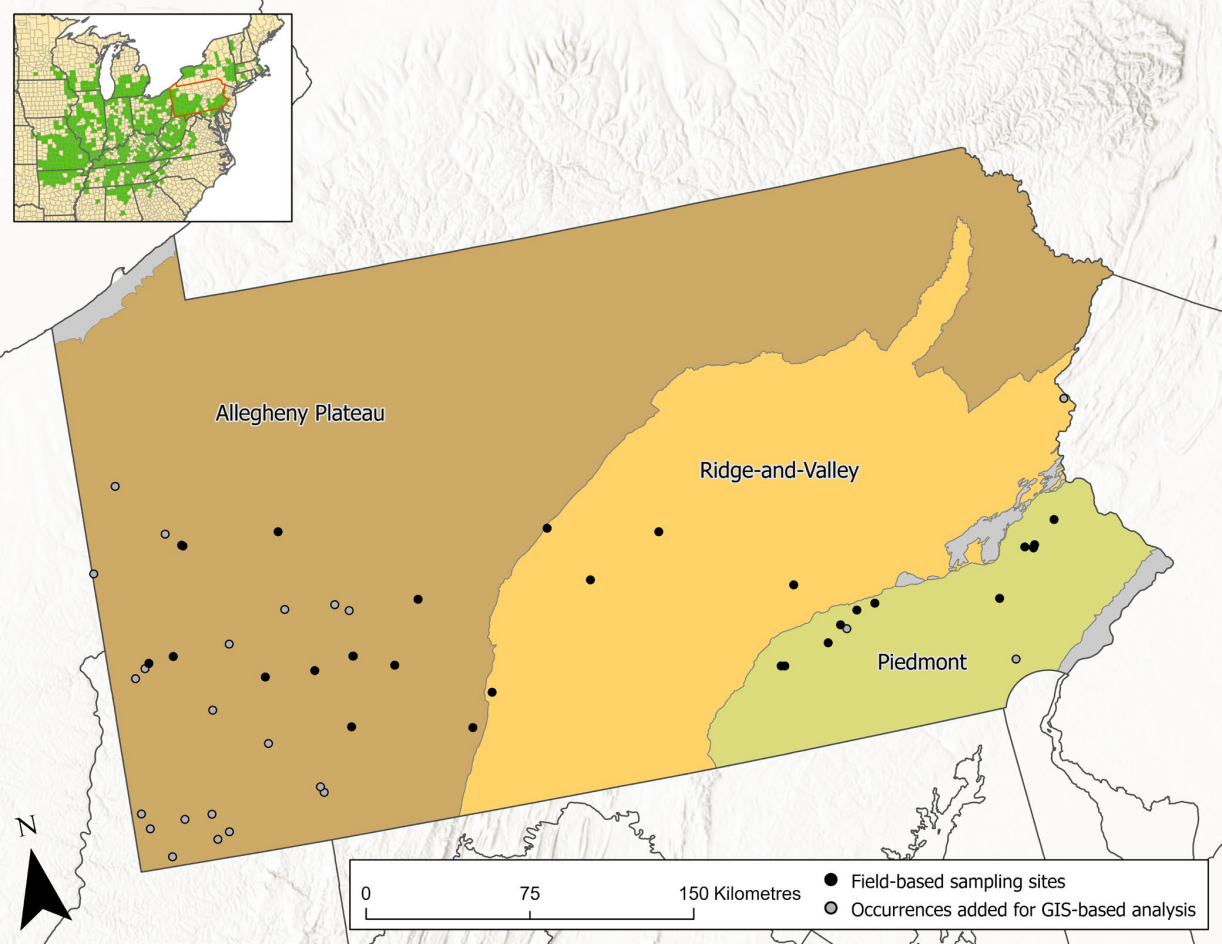
Goldenseal sampling sites and occurrences added for GIS‐based analysis across Pennsylvania.

Goldenseal ( *Hydrastis canadensis* L., Hydrastidaceae/Ranunculaceae) is a slow‐growing herbaceous perennial plant native to eastern North America. It is one of the most important wild‐harvested medicinal plants in the United States (US) (American Herbal Products Association 2020; Kruger et al. 2020) but is listed as “vulnerable” on the International Union for Conservation of Nature (IUCN) Red List of Threatened Species and is included in Appendix II of the Convention on International Trade in Endangered Species of Wild Fauna and Flora (CITES) due to concerns of over‐harvesting for commerce and decline in the wild (Oliver 2017; CITES 2024). The native range of goldenseal spans from southern Ontario, Canada to Kansas, and as far south as Mississippi (Figure 1). Historically, it has been considered most common within its “core range” of Ohio, Indiana, Kentucky, and West Virginia (Lloyd and Lloyd 1884; Sinclair and Catling 2000; NatureServe 2024). The species is less common at the northern edge of its range where it is listed as “Critically Imperiled” (S1) in Vermont, Massachusetts, and Connecticut; and “Imperiled” (S2) in New York and Ontario (NatureServe 2024).

Goldenseal is most frequently reported from rich mesic hardwood forests (NatureServe 2024), but habitat conditions associated with goldenseal can be broad. Forest types in which it has been documented range from dry‐mesic calcareous sites supporting a diverse mix of hardwoods to cove forests in moist lowland sites (Mueller 2004), oak‐hickory forests in the Midwest (Eichenberger and Parker 1976), and sugar maple‐basswood forests in New York (Tait 2006). The broad habitat characteristics associated with goldenseal are notable because it is uncommon in parts of its range, and as a result, researchers have struggled to identify what factors influence its distribution (McGraw et al. 2003; Sanders 2004; Sanders and McGraw 2005).

Given the long‐standing economic and cultural importance of goldenseal, it is possible that the current distribution is heavily influenced by humans (Lloyd and Lloyd 1884; Van Fleet 1914). Records of medicinal use and collection by European settlers date back to the 1700s, and its medicinal role for the Cherokee, Catawba, and other Native American groups dates back further (Hobbs 1990). It is believed that goldenseal has declined across its native range due to ongoing exploitation by commercial harvesters and loss of forested habitat (Mulligan and Gorchov 2004; Sanders 2004; Predny and Chamberlain 2005; Sanders and McGraw 2005; Albrecht and McCarthy 2006; Oliver 2017; NatureServe 2024). Declines attributed to these combined influences were noted over a century ago (Lloyd and Lloyd 1884), and commercial harvest volumes in recent years remain high, ranging from 5006 to 25,604 kg per year between 2011 and 2017 (American Herbal Products Association 2020). Additionally, forested habitats in the eastern US increasingly face challenges associated with land‐use conversion, fragmentation (Riitters et al. 2012), non‐native insects, and diseases (Herms and McCullough 2014). These factors drive changes in species composition, including transitions from native to non‐native dominated plant communities (Harper et al. 2005), which have been found to decrease growth and seed set of native perennial forest herbs and are now pervasive within natural areas in the eastern US (Miller and Gorchov 2004; Miller et al. 2021).

Pennsylvania (PA) is a US state within the northeastern range of goldenseal. Here, the species is designated “Apparently Secure” (S4/G3) (NatureServe 2024), but with a special designation of “vulnerable” (i.e., in danger of population decline within this Commonwealth because its economic value suggests that persons may seek to remove the species from its native habitats) (17 Pa. Code § 45.15.). In PA, the species has been documented primarily in the southwestern and southeastern regions of the state, only rarely in the southcentral Ridge‐and‐Valley Province, and is entirely absent from the northern third of the state. Many populations in PA are large and vigorous but are often separated by large distances. Given its seemingly broad habitat requirements, the reason for this scattered distribution in PA remains poorly understood. Commercial collection of goldenseal has historically focused on “core range states” rather than PA, indicating that overexploitation may not be the primary driver of its low abundance in the state (Zuiderveen 2019). Instead, the legacy of land‐use conversion and habitat fragmentation may play a more important role in limiting its distribution. Many early records of goldenseal were reported in the Piedmont region of southeastern PA (Pennsylvania Natural Heritage Program 2023), but this region contains some of the most fertile soils in the state and has largely been cleared for agriculture (Dewitz 2021). Goldenseal's distribution is further complicated in PA because it is near the northern end of its range. While it occurs sporadically northwards, it is mostly restricted to the Hudson River valley and near Lake Ontario (Tait 2006), where winter temperatures are more moderate than in northern PA due to the influence of the Atlantic Ocean and Great Lakes region (Changnon Jr. and Jones 1972; Fick and Hijmans 2017). It may be the case that PA represents marginal habitat for goldenseal, and that its rarity in the state is due to cold winter temperatures, especially in northern and higher elevation regions.

Although anthropogenic impacts have negatively affected goldenseal (Koffler and Gorby 1957; Lloyd and Lloyd 1884), conservation and husbandry practices have the potential to help the species. One such practice, assisted migration, has been proposed for species in range‐edge locations or where habitat fragmentation limits dispersal ability. This strategy involves establishing plants outside of their existing range in anticipation of changing environmental conditions (Vitt et al. 2010). Existing goldenseal populations can also be positively impacted through in situ conservation efforts. It has been suggested that habitat optimization and population augmentation are required for goldenseal recovery, and altering habitat conditions through natural disturbance simulation has been shown to increase plant size, flowering, and fruit production (Sinclair and Catling 2001; Sinclair et al. 2005). The agroforestry practice of forest farming is another strategy well suited to goldenseal conservation as it leverages the economic value of the species. Forest farming combines the establishment of new patches with habitat optimization to increase yields, potentially accomplishing the same objectives as assisted migration and in situ conservation. Forest farming is of interest to landowners in the state (Strong and Jacobson 2006; McLain and Jones 2013) and goldenseal is already cultivated using this approach in PA (Burkhart, personal observation; Zuiderveen 2019). Successful adoption of forest farming could sustainably meet consumer demand while providing economic livelihood opportunities to those living in forested landscapes (Chittum et al. 2019).

The ability to recognize supportive goldenseal habitat is an important step in these proactive conservation efforts. Habitat modeling through a combination of field and GIS‐based approaches can build an understanding of goldenseal's realized niche and guide site selection for establishing new populations of the species. Specifically, identifying “indicator species” (i.e., nearest neighbors and associates) can inform on‐the‐ground assessments of suitability (Burkhart 2013), since forest vegetation is in part a result of the underlying climatic, topographic, and edaphic factors (Gilliam 2014). However, indicator species are often inadequate predictors of site quality when used alone (Turner and McGraw 2015). For this reason, other field‐based data such as edaphic characteristics (pH, soil nutrients) and GIS‐based habitat suitability modeling can provide broader guidance. GIS‐based habitat suitability modeling can be used to narrow the breadth of potential sites, after which indicators can inform on‐the‐ground assessments and decision‐making (Ren et al. 2010).

Goldenseal presence data are limited by land access and a lack of botanical surveys across the state. Similarly, absence data is complicated by historical and contemporary influences that are difficult to account for. For example, the absence of goldenseal at a site could be the result of previous harvest activities or land use legacies (Bellemare et al. 2002) rather than habitat influences. For this reason, traditional presence/absence modeling is not well suited, and traditional stratified random sampling has limited application (McGraw et al. 2003). Under this context, utilizing a modeling approach that only requires presence data is more suitable. One commonly used method, Maximum entropy (Maxent) (Phillips et al. 2006) outperforms other presence‐only as well as presence/absence modeling techniques such as GLM (Hirzel and Guisan 2002), GAM (Yee and Mitchell 1991), and BIOCLIM (Busby 1991), to differentiate between sites where a species is present and those where it is absent (Elith et al. 2006). This high performance may stem from its ability to fit complex responses and select a relevant set of variables, making it effective when the presence data are limited (Elith et al. 2006; Hernandez et al. 2006; Wisz et al. 2008; Razgour et al. 2011; Yi et al. 2016).

This study combined Maxent modeling incorporating 51 occurrences with community analysis, soil samples, and topographic data collected in the field at 28 sites to provide an understanding of potential goldenseal habitats in the state, and more broadly in the northern edge of its native range. Given the conservation status of the species in this region, results may aid in population discovery and habitat conservation and provide guidance for human interventions such as forest farming and assisted migration in northeastern North America. In undertaking this study, we asked the following questions:

1. What abiotic factors are associated with goldenseal occurrences throughout PA?
2. How do factors encountered in the field compare with those identified by Maxent?
3. What flora is associated with goldenseal occurrences, and which species might be useful for site selection for in situ conservation actions?

## 2 Materials and Methods

### 2.1 Study Area

This study was conducted in PA, within the northeastern US (39°43′–42°16′N; 74°41′–80°31′W). The transition from numerous historic and extant goldenseal occurrences in the southern part of the state to no known occurrences in the northern third of the state creates an opportunity to examine the influence of climate on goldenseal occurrence. Further, PA has a variety of physiographic provinces, including the Allegheny Plateau, Ridge‐and‐Valley, and Piedmont (Shultz 1999), providing an opportunity to identify the influence of edaphic and topographic factors on habitat suitability. Finally, there has been more than three centuries of human influence in PA through logging, habitat conversion, and the introduction of non‐native “invasive” species (DeCoster 1995).

### 2.2 GIS‐Based Methods

#### 2.2.1 Modeling Habitat Suitability

Maxent (version 3.3.3 k) was fit using the “dismo” package (Hijmans et al. 2017) in R Version 4.2.2 (R Core Team 2020). Maxent samples the landscape—defined by environmental covariates—with a set of occurrence points and a set of randomly produced background points. The resulting probability distributions of presences and background points are compared and minimized, outputting an estimated relative occurrence rate based on the principle of maximum entropy (Elith et al. 2011; Yackulic et al. 2013). Specific to this study, a probability distribution for goldenseal habitat suitability was developed using a set of presences mapped against 9881 background points randomly distributed across PA. These background points represent the default number of 10,000 background points for Maxent, with 119 points removed where one or more covariates had missing values due to water features and areas modified by human infrastructure. Predictive performance for Maxent has been shown to be similar across a range of background point levels (Hysen et al. 2022), but the default number of 10,000 has been shown to maximize predictive performance for an area similar to the size of PA in some cases (Phillips and Dudík 2008). Only linear and quadratic features were used in modeling because the primary purpose was to distill abiotic drivers and infer their relative importance on goldenseal habitat rather than maximize predictive accuracy of presences (Merow et al. 2013). Regularization settings were kept as default (Phillips and Dudík 2008).

#### 2.2.2 Occurrence Data Inclusion Criteria

The occurrence data for the habitat suitability model included 51 presences distributed across the known range of goldenseal in PA (Figure 1). Occurrence data was obtained from multiple sources, including field sampling sites (*n* = 28), sites which were found opportunistically in the years following field sampling (*n* = 6), and occurrence points obtained from the Pennsylvania Natural Heritage Program (PNHP) database (*n* = 17). In 2023, the PNHP database included 237 goldenseal occurrences spanning from the 19th century to the present, representing all documented populations in the state. PNHP occurrences were chosen based on a set of selection criteria to ensure consistent data quality between PNHP occurrence data and sites which were visited in the field. First, 163 occurrences which had not been visited since 2012 or were not associated with a named observer were omitted as a proxy to eliminate sites which may have been misidentified or extirpated due to changes in land use or poaching. An additional 35 sites with a location uncertainty distance > 100 m were eliminated, ensuring that occurrence points were within or adjacent to raster cells containing the “true” environment data values corresponding to the populations they represented. Lastly, to reduce the effects of spatial autocorrelation, 22 occurrences located within 2.5 km of one another or located outside the extent of environmental variable raster layers were eliminated. Field‐visited sites and occurrence points with a lower location uncertainty distance were prioritized for retention during the spatial filtering process.

#### 2.2.3 Environmental Predictor Variables

The predictive environment was characterized using 49 environmental variables (Appendix S1: Online Resource 1). These variables represent climate, soils, and topography, and have been used in the US Forest Service Tree Atlas (Peters et al. 2020), DISTIB‐II (Iverson et al. 2019a, 2019b), and SHIFT (Iverson et al. 2019a, 2019b) to model the distributions of 125 tree species in the eastern US. Climatic predictors representing annual trends, seasonality, and extremes of temperature and precipitation from 1970 to 2000 at 1 km resolution were obtained from WorldClim^1^ (Fick and Hijmans 2017). Although annual temperatures have increased in PA since this timeframe, goldenseal is a long‐lived perennial that experiences low rates of seed germination and dispersal (Sanders and McGraw 2005), and without human assistance is likely to lag behind the rapid pace of climate change. Therefore, a more current climate dataset has the potential to skew the modeled climate response away from its “true” suitability. Edaphic features representing physical and chemical properties from the topsoil (0 to 30 cm in depth) and bedrock geology were derived from the 10 m resolution Gridded Soil Survey Geographic^2^ (gSSURGO) dataset using the gSSURGO ArcToolbox in ESRI ArcMap 10.8 by aggregating soil characteristics for each 10 m pixel from the corresponding map unit key (MUKEY) (Soil Survey Staff 2014). Soil variables were aggregated to a depth of 30 cm to maintain consistency in edaphic representation across soil types with a range in profile depths. The integrated moisture index was developed by Iverson et al. (1997) and other topographic variables including aspect (Beers et al. 1966), roughness (Riley et al. 1999), and topographic position indices (Weiss 2001) were derived from a 1 m digital elevation model (DEM) obtained from the PAMAP program^3^ (Pennsylvania Department of Conservation and Natural Resources 2006). Goldenseal populations often cover areas greater than 1 m or 10 m. To better link edaphic and topographic predictor variables to the range of conditions within the extent of goldenseal populations, variables were resampled to a 90 m resolution. Edaphic predictor variables were resampled by calculating averages over 3 × 3 10 m pixels using ESRI ArcMap 10.8. For topographic variables, the 1 m DEM was first resampled to 90 m resolution, and all topographic variables were calculated from the resulting DEM.

After preliminary analysis, the seven most influential variables were selected for the final model to reduce overfitting and aid in interpretability (Table 1). These variables were selected stepwise based on their pairwise Pearson correlation coefficients, permutation importance, variable dependency, and biological interpretability (Appendix S1: Online Resource 2). First, a Maxent model was fitted using all variables, and permutation importance was obtained for all variables. Pearson correlation coefficients between all variables were computed using the “ENMTools” package in R (Warren et al. 2021). Correlated variables were eliminated based on a threshold of 0.7 or greater (i.e., exhibiting high collinearity), retaining the one with the higher permutation importance in the full model for each pair of correlated variables. All variables with a permutation importance of 0 were eliminated regardless of correlation. Finally, the variables whose marginal response curves showed dependency upon other variables (i.e., a change in response curve between full model and single variable model) were eliminated to aid in the interpretation of biological significance.

**TABLE 1:** Descriptions, contributions, and sources of predictor variables used to develop the goldenseal habitat suitability model in Maxent.

Variable	Description	Percent contribution	Source
BIO11	Mean temperature of the coldest quarter (°C)	30.5	Fick and Hijmans (2017)
BEDROCK	Bedrock type	52	PA Department of Conservation and Natural Resources
OM	Organic matter content (% by weight)	3.2	USDA Natural Resource Conservation Service
Permeability	Soil permeability rate (cm/h)	0.3	USDA Natural Resource Conservation Service
SOILSUBORD	Soil suborder based on USDA soil taxonomy	10.4	USDA Natural Resource Conservation Service
ERR15	Elevation relief ratio (15‐pixel neighborhood)	3.1	PA Department of Conservation and Natural Resources
IMI	Integrated Moisture Index	0.4	Iverson et al. (1997)

#### 2.2.4 Model and Variable Evaluation

The model performance was evaluated using two metrics commonly used in habitat suitability modeling: area under the receiver operating characteristic curve (AUC) and continuous Boyce index (CBI) (Feng 2023). AUC is a popular model evaluation metric in the Maxent literature and is interpreted as the probability that a randomly chosen occurrence location is ranked higher than a randomly chosen background point (Merow et al. 2013). The AUC metric is a default Maxent output and ranges from 0 to 1, with values above 0.5 for models with predictive ability better than random and with 1.0 for those with perfect predictive ability (Phillips et al. 2006). AUC has been criticized as inadequate when used to evaluate presence‐only models because it does not directly quantify overfitting (Radosavljevic and Anderson 2014) and undervalues models that do not provide predictions across the entire spectrum of proportional areas in the study area (Peterson et al. 2008). However, AUC does have advantages over other evaluation tools such as the true skill statistic (TSS) because it is threshold‐independent (Peterson et al. 2008). Uncertainty in the AUC metric was evaluated using k‐fold cross‐validation. Due to the relatively small sample of occurrence points (*n* = 51), *k* = 5 was chosen to limit the chance of spatially correlated folds. For each subset, the model was trained with *k*−1 subsets and tested on the *k*th subset (Merow et al. 2013). In effect, the 51 occurrence points were randomly partitioned into 5 subsets, and the model was tested on each subset after using the other subsets to train the model. Assuming an unbiased sample of occurrence points, cross‐validation is a useful metric for model performance because it enables comparison between training AUC and testing AUC with independent occurrence data (Araújo et al. 2005). CBI was calculated using the “ecospat” package in R with default settings (Di Cola et al. 2017). Like AUC, CBI is a threshold‐independent metric. However, CBI has the advantage of evaluating model performance based on presences alone, making it more appropriate for models without true absence data, as well as providing predicted‐to‐expected ratio (P/E) curves that offer further insights into the model's ability to distinguish between different classes of suitability. CBI indicates the level at which a model's predictions are consistent with the presence's distribution in the evaluation dataset, ranging from −1 to 1, with positive values indicating better performance than random (Hirzel et al. 2006).

Variable importance was estimated using a jackknife test by excluding each variable in turn, fitting a model with the remaining variables, and subsequently fitting a model with each variable in isolation (Phillips et al. 2006). Variable importance was then evaluated based on model performance when individual variables were excluded or used in isolation in comparison to the model fitted with the other six variables. The relationship between species presence and each of the seven variables was interpreted based on their response curves. These curves show how the logistic prediction (interpreted as relative habitat suitability on a scale of 0–1) changes across values for each variable.

### 2.3 Field Methods

#### 2.3.1 Goldenseal Population Determination

During 2015 and 2016, wild population occurrences were solicited from botanists, root diggers, forest landowners, and through examination of herbarium specimens. A focused effort was made to solicit new sites from regions of the state where goldenseal had never been recorded to determine for certain whether the species occurred in these regions but had been missed to date. This was done by sharing news and solicitations via media (including social media) and by having conversations with experienced root diggers and buyers familiar with the plant and the region.

At each study site, up to four sampling plots were established within goldenseal occurrences. Plots were arranged to capture any edaphic, topographic, and floristic variation within each site. Goldenseal is a clonal species, spreading through rhizome expansion to form discreet colonies separated by gaps (Sanders 2004; Christensen and Gorchov 2010). Plot spacing and number varied according to population spread and the number of discrete occurrences, with the objective of documenting only vegetation nearest to and interspersed within each goldenseal colony. Populations varied in size from thousands of ramets spread over multiple hectares to single colonies of approximately 100 ramets. All populations showed evidence of sexual reproduction (i.e., flowering and fruit set).

A total of 58 plots were established at 28 field sites for community analysis. Study sites were in the southern two‐thirds of the state within 19 counties, including two counties in which goldenseal had not previously been recorded. Eleven sites were located on the Allegheny Plateau, five in the Ridge‐and‐Valley, and 12 in the Piedmont province (Figure 1).

#### 2.3.2 Field Site and Community Sampling

At each site, topographic position, aspect, and a short description were recorded, and population sizes and areal extents were estimated. Soil samples were collected from the upper 10 cm (A‐horizon) in each plot after large coarse organic matter was removed. Samples were analyzed by the Penn State Agricultural Analytical Services Lab. Results included soil texture as well as cation exchange capacity (CEC), water pH, phosphorus, potassium, magnesium, and calcium determined by Mehlich‐3 (ICP). Soil samples were collected from all sites, but texture was only analyzed at a subset of sites (*n* = 19, 65% of sites) due to limited financial resources.

Overstory dominant and co‐dominant trees were recorded in each plot using the point‐centered quarter method (PCQM); the nearest tree species from the plot center in each quarter was recorded for a total of four trees per plot. For each tree, the distance from the plot center and diameter at breast height (DBH) were recorded. An inventory of understory vegetation (shrubs, ferns, and herbaceous species) was conducted within a circular 0.01‐ha plot. Each plot was visited twice during the spring and summer of 2016 and 2017 to capture seasonal changes in associated flora. Voucher specimens of goldenseal were collected from each field site and deposited at the Pennsylvania State University Herbarium (PAC), the Carnegie Museum of Natural History Herbarium (CM), and the Morris Arboretum of the University of Pennsylvania Herbarium (MOAR).

#### 2.3.3 Statistical Analysis of Field Data

Descriptive statistics were generated for floristic and edaphic data from each plot. Indicator species analysis (ISA) (McCune and Grace 2002) was used to determine differences in associated species based on physiographic province, soil calcium content, and pH. Thresholds for edaphic factors were based on guidelines to maintain a mixed‐species woodlot as provided by the Pennsylvania State Soil Analytics Lab. Significance was determined using a Monte Carlo randomization procedure with 4999 randomizations (McCune and Grace 2002). All ISA analyses were performed using PC‐ORD (McCune and Mefford 2011). Additionally, importance value percentages were calculated on dominant and co‐dominant tree species based on relative frequency, relative density, and relative dominance (Curtis and McIntosh 1951).

## 3 Results

### 3.1 Maxent Model Performance

**FIGURE 2:**
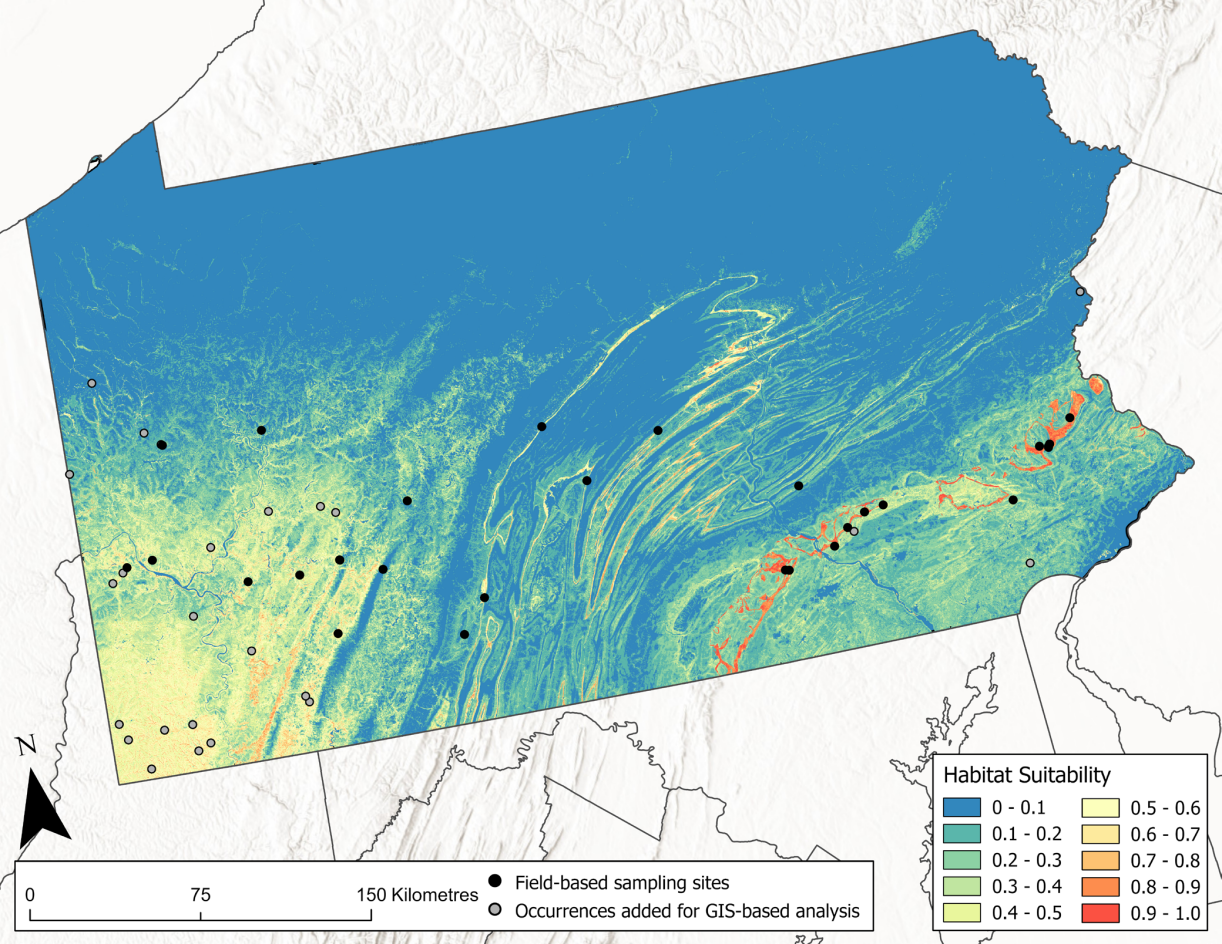
The Maxent model projected onto the environmental variables with occurrence points. Warmer colors show areas with a higher predicted suitability for goldenseal.

**FIGURE 3:**
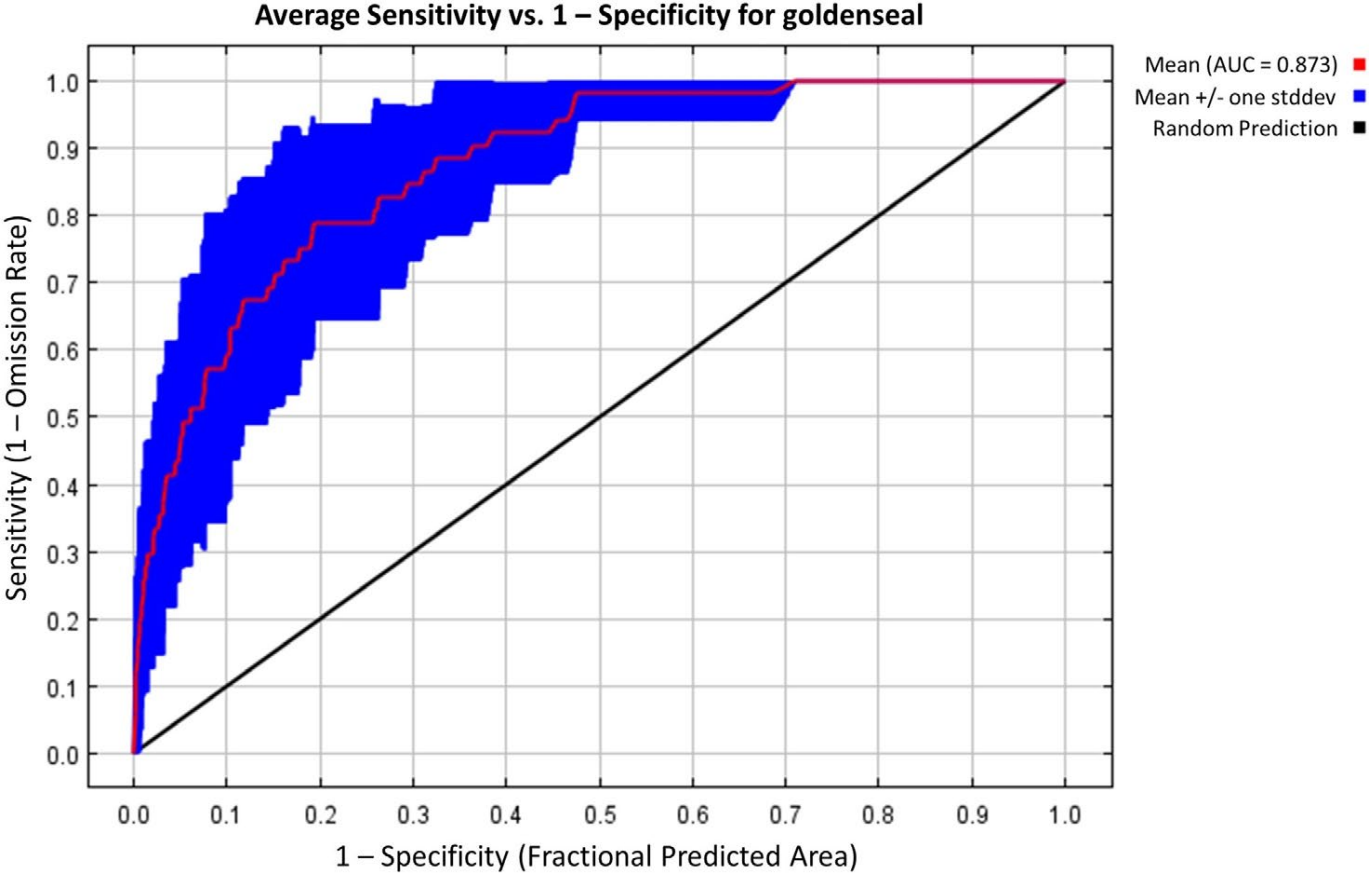
Habitat suitability model test AUC curve for goldenseal. Specificity is established using predicted area, rather than true commission due to having presence presence‐only data.

**FIGURE 4:**
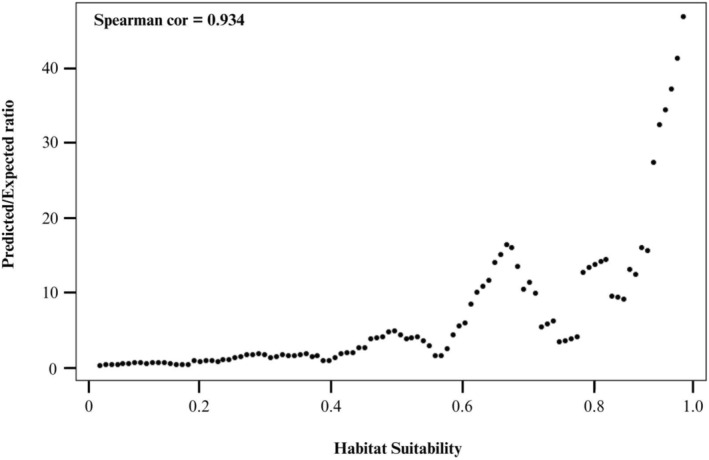
Spearman correlation coefficient and P/E ratio plots for the continuous Boyce Index.

One climatic, four edaphic, and two topographic variables were used to fit the final model and project habitat suitability across PA (Table 1, Figure 2). The resulting model had good predictive ability to differentiate between suitable and unsuitable habitat, with a training AUC of 0.903. Replicate cross‐validation runs had an average testing AUC of 0.873, with a standard deviation of 0.063 (Figure 3). CBI showed a Spearman correlation of 0.934 (Figure 4), also suggesting a high degree of predictive ability. P/E ratios increased as habitat suitability increased, except for regions with habitat suitability between 0.5–0.6 and 0.7–0.8 (Figure 4).

### 3.2 Climatic Results

**FIGURE 5:**
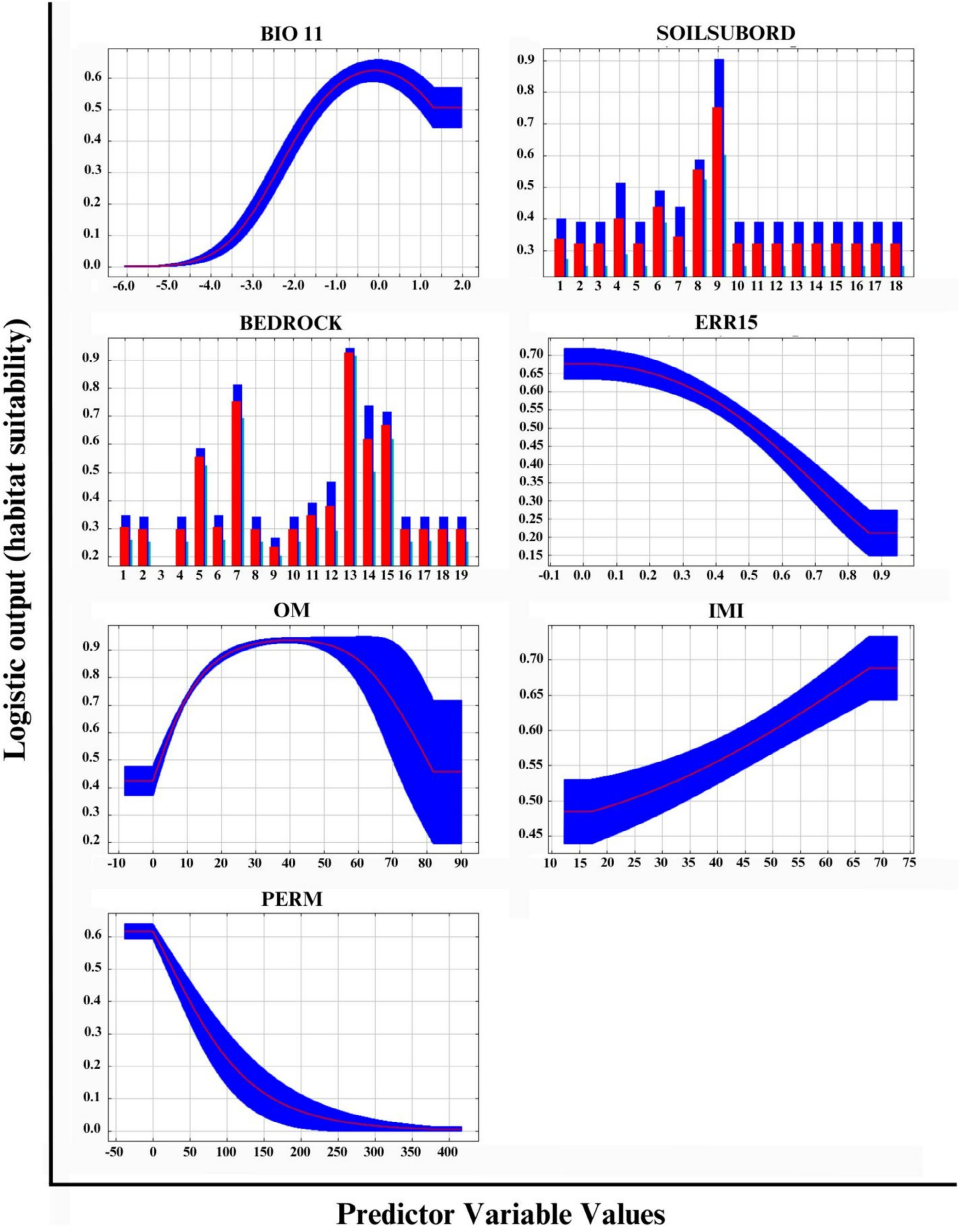
Marginal Response curves for the 7 predictor variables included in the final goldenseal habitat suitability model. An explanation of each variable can be found in Table 1.

Mean temperature of the coldest quarter (i.e., the average winter temperature) was the only climatic variable included in the final model, but of second highest importance with a percent contribution of 30.5% (Table 1). Suitability increased as average winter temperature increased, peaking at 0.0°C, near the upper end of average winter temperatures in PA. Suitability decreased with decreasing winter temperatures down to −5.4°C, near the lower limit of average winter temperatures in the state (Figure 5).

### 3.3 Edaphic Results

Bedrock type was the most important modeled predictor variable with a percent contribution of 52% (Table 1). Suitable types differed across the state according to physiographic province: Pennsylvanian and Permian formations on the Allegheny plateau, Devonian and Silurian limestone in the Ridge‐and‐Valley, and Jurassic diabase in the Piedmont. Soil suborder was also influential, with aquents and udults showing the highest suitability among suborders and a percent contribution of 10.4% (Figure 5, Table 1). Organic matter content and permeability rate were influential to a lesser degree, with percent contributions of 3.2% and 0.3%, respectively (Table 1). Organic matter showed a unimodal response, with habitat suitability peaking at 40% OM content, while soil permeability was inversely related to habitat suitability (Figure 5). Soil pH and calcium levels were high on average (6.3 and 1778 ppm respectively), but macronutrients varied considerably, with standard deviations commonly half the value of the mean (Table 2). Soil texture also varied, but loam soils were most common (Table 2).

**TABLE 2:** Soils summary data from goldenseal populations in Pennsylvania.

Nutrients^a^	Mean (SD)	Range	Texture^b^	Mean (SD)	Range
pH	6.3 (0.6)	5.0–7.3			
P (ppm)	21 (16)	4–85			
K (ppm)	215 (70)	78–351	% Sand	50.0 (10.1)	35.0–69.3
Mg (ppm)	242 (119)	60–600	% Silt	32.7 (7.2)	15.0–39.2
Ca (ppm)	1778 (866)	400–5040	% Clay	17.3 (7.0)	7.9–32.8

^a^ *n* = 58.

^b^ *n* = 19.

### 3.4 Topographic Results

The model identified two topographic variables that were influential to goldenseal habitat suitability. Elevation‐relief ratio (ERR15), which serves as an indicator of landscape position, contributed 3.1% to the overall model (Table 1), with lower values of ERR15 (lower slope positions) predicting higher habitat suitability (Figure 5). Integrated Moisture Index (IMI) was less influential, with a percent contribution of 0.4% (Table 1), and showing high predicted suitability at high moisture levels (Figure 5).

### 3.5 Community Analysis Results

Forest community types where goldenseal was found included “Tuliptree‐Beech‐Maple,” “Red Oak‐Mixed Hardwood,” and “Central Appalachian Rich Cove” (NatureServe 2022; Zimmerman and Hnatkovich 2022; Zimmerman and Fike 2022). A total of 159 species were documented in the study plots: 24 overstory trees; 20 vines, shrubs, and understory trees; 115 herbaceous plants. Of these species, 11% (*n* = 17) were non‐native (Appendix S1: Online Resource 4).

The most common tree associates were tulip‐poplar ( *Liriodendron tulipifera* ) and sugar maple ( *Acer saccharum* ), which occurred in 40% and 38% of plots, respectively, and had the highest importance value percentages among associated tree species (Tables 3, 4). The most common shrub was spicebush ( *Lindera benzoin* ), which occurred in 83% of the plots (Table 5). The most common herbaceous species was Jack‐in‐the‐pulpit ( *Arisaema triphyllum* ), which occurred in 79% of the plots. Rattlesnake fern (*Botrypus virginianus*) and marginal wood fern ( *Dryopteris marginalis* ) were the most common ferns, occurring in 55% of plots. Of the 39 herbs and ferns that were present in more than 20% of plots, 13 differed according to physiographic province, three to calcium levels, and eight to pH (Table 6).

**TABLE 3:** Relative abundances and importance value percentages (IV %) for dominant or co‐dominant overstory tree species associated with goldenseal in Pennsylvania.

Scientific name^a^	Common name	Relative abundance			IV%
		Frequency	Density	Dominance	
*Liriodendron tulipifera* L.	Tulip‐poplar	15.4	21.4	32.4	23.1%
*Acer saccharum* Marshall	Sugar maple	14.8	21.4	12.6	16.2%
*Juglans nigra* L.	Black walnut	9.4	7.0	8.3	8.2%
*Quercus rubra* L.	Northern red oak	6.7	5.2	11.5	7.8%
*Quercus alba* L.	White oak	6.0	6.1	4.8	5.6%
*Carya cordiformis* (Mill.) K. Koch	Bitternut hickory	6.0	4.8	3.4	4.7%
*Prunus serotina* L.	Black cherry	4.7	3.5	4.0	4.1%
*Carya ovata* (Mill.) K. Koch	Shagbark hickory	4.0	3.9	3.1	3.7%
*Carya glabra* (Mill.) Sweet	Pignut hickory	4.7	3.5	2.3	3.5%
*Carya tomentosa* (Poir.) Nutt.	Mockernut hickory	3.4	3.1	3.1	3.2%
*Tilia americana* L.	American basswood	4.7	3.1	1.5	3.1%
*Acer platanoides* L.	Norway maple	2.0	3.1	3.5	2.9%
*Fraxinus americana* L.	White ash	2.7	2.2	2.8	2.6%
*Ulmus rubra* Muhl.	Slippery Elm	2.7	2.2	1.5	2.1%
*Fraxinus pennsylvanica* Marshall	Green ash	2.0	1.3	1.2	1.5%
*Quercus montana* Willd.	Chestnut oak	2.0	1.3	0.7	1.4%
*Pinus strobus* L.	Eastern white pine	2.0	0.9	1.1	1.3%
*Magnolia acuminata* L.	Cucumber tree	1.3	1.3	0.5	1.0%
*Fagus grandifolia* Ehrhart	American beech	1.3	0.9	0.8	1.0%
*Fraxinus nigra* Marshall	Black ash	1.3	0.9	0.7	1.0%
*Robinia pseudoacacia* L.	Black locust	0.7	1.7	0.2	0.9%
*Picea abies* (L.) H. Karst.	Norway spruce	0.7	0.4	0.5	0.5%
*Prunus avium* L.	Bird cherry	0.7	0.4	0.5	0.5%
*Tsuga canadensis* (L.) Carrière	Eastern hemlock	0.7	0.4	0.3	0.5%

^a^ All taxonomy follows Weakley (2023).

**TABLE 4:** Dominant and co‐dominant trees associated with goldenseal in Pennsylvania along with indicator species analysis (ISA) results.

Scientific name^a^	Common name	% of plots and (*n*)	ISA variables		
			Prov	Ca	pH
*Liriodendron tulipifera* L.	Tulip‐poplar	40 (23)	P**		AO**
*Acer saccharum* Marshall	Sugar maple	38 (22)		AO*	O*
*Juglans nigra* L.	Black walnut	22 (13)	RV**		
*Carya cordiformis* (Mill.) K. Koch	Bitternut hickory	16 (9)			AO**
*Quercus alba* L.	White oak	16 (9)			
*Quercus rubra* L.	Northern red oak	16 (9)			
*Carya glabra* (Mill.) Sweet	Pignut hickory	14 (8)	P***		AO***
*Prunus serotina* L.	Black cherry	12 (7)	AP**		
*Tilia americana* L.	American basswood	10 (6)			
*Carya tomentosa* (Poir.) Nutt.	Mockernut hickory	9 (5)	P**	O*	AO**
*Carya ovata* (Mill.) K Koch	Shagbark hickory	9 (5)			
*Ulmus rubra* Muhl.	Slipper elm	9 (5)			
*Acer platanoides* L.*	Norway maple	5 (3)	AP**		
*Fraxinus pennsylvanica* Marshall	Green ash	5 (3)			AO*
*Quercus montana* Willd.	Chestnut oak	5 (3)			
*Fagus grandifolia* Ehrhart	American beech	5 (3)			

*Note:* Only associates occurring on 5% or more research plots are given (*n* = 58 plots). Monte Carlo test of significance *p*: *p ≤ 0.10, **p ≤ 0.05, ***p ≤ 0.01. Prov: AP, Appalachian Plateau; P, Piedmont; RV, Ridge and Valley. Ca: below optimum (BO) (< 1400 ppm), optimum (O), and above optimum (AO) (> 2100 ppm). pH: below optimum (BO) (< 6.0), optimum (O), and above optimum (AO) (> 7.0). Thresholds for calcium and pH were set based on recommendations by the PSU Agricultural Analytical Services Laboratory for maintaining mixed species woodlots. Asterisks (*) denote non‐native, exotic species.

^a^ All taxonomy follows Weakley (2023).

**TABLE 5:** Shrubs and vines associated with goldenseal in Pennsylvania along with indicator species analysis (ISA) results.

Scientific name^a^	Common name	% of plots and (*n*)	ISA variables		
			Prov	Ca	pH
*Lindera benzoin* L.	Spice bush	83 (44)			
*Parthenocissus quinquefolia* (L.) Planch.	Virginia creeper	81 (43)	RV*		AO*
*Berberis thunbergii* DC.*	Japanese barberry	58 (31)	P***		
*Rosa multiflora* Thunb.*	Multiflora rose	51 (27)	RV*		
*Toxicodendron radicans* (L.) Kuntze	Poison ivy	51 (27)	p**		
*Viburnum acerifolium* L.	Maple‐leaf viburnum	15 (8)			
*Smilax hispida* Muhl. ex Torr.	Bristly greenbriar	13 (7)			
*Vitis spp*.	Wild grape	13 (7)	P*	BO*	
*Lonicera japonica* Thunb.*	Japanese honeysuckle	11 (6)			AO*

*Note:* Only associates occurring on 10% or more research plots are given (*n* = 58 plots). Monte Carlo test of significance *p*‐values: *p ≤ 0.10, **p ≤ 0.05, ***p ≤ 0.01. Prov: AP, Appalachian Plateau; P, Piedmont; RV, Ridge and Valley. Ca: below optimum (BO) (< 1400 ppm), optimum (O), and above optimum (AO) (> 2100 ppm). pH: below optimum (BO) (< 6.0), optimum (O), and above optimum (AO) (> 7.0). Thresholds for calcium and pH were set based on recommendations by the PSU Agricultural Analytical Services Laboratory for maintaining mixed species woodlots. Asterisks (*) denote non‐native, exotic species.

^a^ All taxonomy follows Weakley (2023).

**TABLE 6:** Herbaceous species associated with goldenseal in Pennsylvania, along with indicator species analysis (ISA) results.

Scientific name^a^	Common name	% of plots and (*n*)	ISA variables		
			Prov	Ca	pH
*Arisaema triphyllum* (L.) Schott	Jack‐in‐the‐pulpit	79 (42)	P*		AO*
*Podophyllum peltatum* L.	Mayapple	58 (31)			
*Botrypus virginianus* (L.) Michx.	Rattlesnake fern	55 (29)			AO*
*Dryopteris marginalis* (L.) A. Gray	Marginal wood fern	55 (29)	RV**		
*Amphicarpa bracteata* (L.) Fernald	American hogpeanut	51 (27)			
*Actaea racemosa* L.	Black cohosh	47 (25)	RV***		
*Microstegium vimineum* (Trin.) *A. Camus* *	Japanese stiltgrass	47 (25)	RV*		
*Polystichum acrostichoides* (Michx.) Schott	Christmas fern	45 (24)	RV*		
*Solidago/Symphiotrichum spp*.	Goldenrod/aster	45 (24)			
*Uvularia perfoliata* L.	Perfoliate bellwort	45 (24)			
*Galium circaezens* Michx.	Licorice bedstraw	43 (23)			
*Viola pubescens* Ait.	Downy yellow violet	43 (23)	P***		
*Geranium maculatum* L.	Wood geranium	42 (22)			
*Viola hirsutula* Brainerd	Southern woodland violet	42 (22)	P*		
*Alliaria petiolata* (M. Bieb.) Cavara & Grande*	Garlic mustard	41 (21)			
*Circaea canadensis* L.	Enchanter's nightshade	41 (21)			
*Sanicula spp*. L.	Black snakeroot	40 (21)	AP*	AO*	
*Asarum canadense* L.	Wild ginger	36 (19)			
*Persicaria longiseta* (Bruijn) Kitagawa*	Bristled knotweed	36 (19)	RV**		
*Sanguinara canadensis* L.	Bloodroot	36 (19)	P***		
*Maianthemum racemosum* (L.) Link	False Solomon's seal	34 (18)	P***		AO***
*Agrimonia spp*. Tourn. ex L.	Agrimony	32 (17)			
*Osmorhiza claytonii* (Michx.) C.B. Clarke	Clayton's sweetroot	32 (17)			AO**
*Nabalus spp*. Cass.	Rattlesnake root	32 (17)			AO*

*Note:* Only associates occurring on 30% or more research plots are given (*n* = 58 plots). Monte Carlo test of significance *p*‐values: *p ≤ 0.10, **p ≤ 0.05, ***p ≤ 0.01. Prov: AP, Appalachian Plateau; P, Piedmont; RV, Ridge and Valley. Ca: below optimum (BO) (< 1400 ppm), optimum (O), and above optimum (AO) (> 2100 ppm). pH: below optimum (BO) (< 6.0), optimum (O), and above optimum (AO) (> 7.0). Thresholds for calcium and pH were set based on recommendations by the PSU Agricultural Analytical Services Laboratory for maintaining mixed species woodlots. Asterisks (*) denote non‐native, exotic species.

^a^ All taxonomy follows Weakley (2023).

Some associates indicated regional or edaphic site conditions. In the Piedmont, ISA identified tulip‐poplar, pignut hickory ( *Carya glabra* ), and mockernut hickory ( *Carya tomentosa* ) to be important overstory components, with Jack‐in‐the‐pulpit, downy yellow violet ( *Viola pubescens* ), southern woodland violet ( *Viola hirsutula* ), bloodroot ( *Sanguinaria canadensis* ), and false Solomon's seal ( *Maianthemum racemosum* ) in the understory. In the Ridge and Valley province, black walnut ( *Juglans nigra* ) was an indicator in the overstory, with marginal wood fern, black cohosh ( *Actaea racemosa* ), and Christmas fern ( *Polystichum acrostichoides* ) in the understory. The Allegheny Plateau had fewer regionally specific associates, but black cherry ( *Prunus serotina* ) was identified as an indicator in the overstory. Indicators of specific edaphic conditions included tulip‐poplar, bitternut hickory ( *Carya cordiformis* ), pignut hickory, mockernut hickory, and green ash (*Fraxinus pensylvanica*) in the overstory, with Jack‐in‐the‐pulpit, rattlesnake fern, false Solomon's seal, hairy sweet cicely (*Ozmorhiza claytonii*), and rattlesnake root (*Nabalus* spp.) in the understory on sites with pH 7 or greater. Sugar maple was an indicator on sites with calcium above 2100 ppm and pH between 6 and 7.

Several non‐native species were also present, including 13 taxa classified as moderately to highly invasive in PA (Appendix S1: Online Resource 4) Of these, at least one was found in 83% of field plots. The most prevalent non‐native taxa were woody shrubs; Japanese barberry ( *Berberis thunbergii* ) and multiflora rose ( *Rosa multiflora* ) both occurred in over 50% of plots (Table 5). Non‐native herbaceous species were also common, with Japanese stilt grass ( *Microstegium vimineum* ) and garlic mustard ( *Alliaria petiolata* ) both occurring in over 40% of plots (Table 6).

## 4 Discussion

The results of this study can aid in situ conservation efforts by suggesting sites for goldenseal introductions and/or forest farming. Model results suggest that suitable goldenseal habitat in PA is governed by bedrock type and winter temperature, but that bedrock may serve as a proxy for land‐use legacy in the model. Low soil acidity and high moisture availability also predicted suitable habitat, traits which were also observed in field sampling and shared by several species within plant communities where goldenseal populations occurred. While the presence‐only methods used in this study limit overreliance on individual site characteristics and plant indicators for determining habitat suitability, GIS‐based habitat suitability modeling and field data provided independent confirmation of environmental conditions influencing goldenseal habitat.

### 4.1 Climatic Site Factors

PA is near the northern edge of goldenseal's native range, with only a limited number of isolated populations further north. Many of these northern populations occur in the Great Lakes region and in the Hudson Valley, where winter temperatures are moderated by the Great Lakes and Atlantic Ocean, respectively, compared to the highlands of northern PA (Christensen and Gorchov 2010; Fick and Hijmans 2017). Goldenseal has been more commonly reported from southwestern PA, which is contiguous with the “core range” extending south through the Ohio river valley (Lloyd and Lloyd 1884; McGraw et al. 2003; Christensen and Gorchov 2010; NatureServe 2024). Climate data used in modeling represents a period ranging from 1970 to 2000. A warming climate in the decades since and the predicted increase in average winter temperatures over the next 50 years (Fan et al. 2015) make exact temperature requirements difficult to determine. It may be the case that goldenseal's absence in northern PA represents a lag in expansion from previously unsuitable climate conditions due to dispersal or other limitations. A warming climate may be favorable for goldenseal, opening previously unsuitable sites to natural or assisted migration and increasing the viability of forest farming in northern PA and other areas northward. However, model results are only correlative, and the influence of climate on goldenseal habitat in PA requires further investigation. Planting studies to determine specific temperature tolerances and models incorporating projected future winter temperatures could assess the degree to which goldenseal habitat may expand in PA and the northeastern US and identify potential areas for forest farming and assisted migration.

### 4.2 Edaphic Site Factors

The Ridge‐and‐Valley province is characterized by a series of valleys underlain by limestone, dolomite, or shale, and ridges comprised of sandstone and quartzite (Shultz 1999). Goldenseal is not prevalent in the region, and only five occurrences were available for modeling. Four of these occurred on narrow bands of Devonian and Silurian limestone comprising the lower slopes below erosion‐resistant sandstone ridges (Berg et al. 1980). The suitability of limestone bedrock is consistent with pH and calcium levels found in soil testing results (Table 2). Although identified by the model as the only suitable bedrock type in the Ridge‐and‐Valley, it may be that this zone represents the margin of goldenseal's original habitat in this region, having been extirpated from more fertile limestone and dolomite valley bottom sites through agricultural conversion (Fletcher 1951). Conversely, despite supporting intact forest habitat, the sandstone and quartzite ridges of the region were identified as less suitable. It is likely that the unsuitability of sandstone bedrock types in the Ridge‐and‐Valley is due to their acidic nature (Blumberg et al. 1982).

The Piedmont region in southeastern PA has some of the most fertile soil in the state and historically supported goldenseal (PNHP 2023). However, most of the forest in this region has been cleared for agriculture. The process of land conversion likely destroyed much of the goldenseal in the Piedmont, as has also been documented in Ohio (Koffler and Gorby 1957). The Gettysburg–Newark lowlands, which are made up of erosion‐resistant Jurassic diabase, are an exception. Diabase creates rocky outcroppings that prevent farming (Blumberg et al. 1982), and areas underlain by this bedrock support rich, relatively intact forests in an otherwise highly fragmented agricultural landscape. Many of the extant goldenseal populations in this part of the state occur over Jurassic diabase due to this land use legacy. A similar trend has been observed in Virginia, where two goldenseal populations were reported growing on sites underlain by otherwise dissimilar bedrock types sharing the characteristic of being unsuitable for farming (Mueller 2004). However, further investigation is needed as to whether the apparent absence of goldenseal on reverted agricultural sites is due to a lack of dispersal or a phase shift impeding recolonization. Additionally, models incorporating land‐use legacy directly may better predict goldenseal's distribution throughout the state.

The influence of bedrock on goldenseal habitat in the southwestern part of the state appears to be tied less to land use legacy than in the Ridge‐and‐Valley or Piedmont, due to the widespread nature of Pennsylvanian and Permian bedrock in this region. Both Pennsylvanian and Permian bedrock are comprised of sequential layers of sandstone, shale, limestone, and coal, and tend to be eroded into steeply sloping hills (Berg et al. 1980). One feature that does stand out as unsuitable for goldenseal habitat is the Mississippian bedrock of the Laurel Highlands. This bedrock is partly composed of erosion‐resistant acidic sandstone and forms some of the highest mountains in the state. While the acidity of Mississippian bedrock may play a role in decreasing habitat suitability, lower winter temperatures at these high elevations are likely important as well.

Soil suborder was also found to influence habitat suitability. Aquents, soils of recent origin that are usually seasonally or permanently inundated, were the strongest predictors of suitable habitat. These findings are partially supported by previous reports indicating that goldenseal grows well in moist conditions, including wet, predominantly sandy or clay soils. However, contrary to model results, well‐drained mesic soils are considered optimal (Penskar et al. 2001; Upton 2001; Sinclair and Catling 2001). The high suitability of Aquents may be a misleading result of the 90 m resolution of the soil layer capturing occurrences growing on lower slopes adjacent to floodplains, but soil moisture measurements were not collected, pointing to a need for more data.

Udults were also suitable, and as with bedrock results, their suitability may be a function of land‐use legacy. Udults are lower in base saturation than other common mesic soil types such as Udalfs and often require soil amendments to be used as cropland. They support a high proportion of forested habitat in the state, having escaped land‐use conversion (Dewitz 2021; Soil Survey Staff 1999). Taken together, soil chemistry and model results indicate that extant populations are disproportionately represented on wet to mesic sites that have escaped land‐use conversion and are not highly acidic but otherwise range broadly in fertility. Given the limitations of the NRCS soil data in representing specific soil macronutrient contents and the fact that soil fertility was only sampled at 28 of 51 occurrences used in modeling, more data may be needed to fully interpret these results.

Less influential edaphic variables included organic matter content and permeability rate, both of which relate to soil moisture (Randall and Anderson 2005). Suitability increased with increasing organic matter and decreased in soils with rapid permeability rates, pointing to wetter soil being more suitable (Figure 5). While soil organic matter was not measured in the field, goldenseal's predilection toward sites with high organic matter is strongly supported by previous literature (Penskar et al. 2001; Upton 2001; Sinclair and Catling 2001; Tait 2006). Modeled suitability of soil permeability rates mostly coincides with textural classes measured in the field, which were predominantly loam. Loam soils have also been reported in previous literature (Penskar et al. 2001) suggesting that goldenseal prefers moderately permeable soils. However, the model also identified impermeable soils to be high in suitability, contradicting both field measurements and literature (Sinclair and Catling 2001; Tait 2006). This response is potentially due to the coarse resolution of the permeability raster being unable to accurately portray a relationship between soil texture and goldenseal suitability.

### 4.3 Topographic Site Factors

Although calculated differently, both topographic variables included in the model were related to soil moisture. ERR15 captures elevation relative to the surrounding topography, while IMI incorporates both soil and topographic features, including slope, aspect, cumulative flow of water downslope, landscape curvature, and soil water holding capacity (Iverson et al. 1997). Despite differences in calculation and relative importance in the model, the suitability curves for ERR15 and IMI point to goldenseal's preference for moist conditions.

Field observations of topography corroborated with the response curves of ERR15 and IMI. Although some populations sampled in the field occurred across a range of aspects and topographic conditions, most occupied moist environments in lower slope or bottomland positions (Appendix S1: Online Resource 3), supporting model results. Goldenseal's affinity for lower slope positions has been documented (Meyer and Parker 2003; Tait 2006), and increased soil moisture has been found to increase goldenseal seedling success and growth (Douglas et al. 2002; Albrecht and McCarthy 2006).

### 4.4 Community Analysis

Forest communities associating with goldenseal in this study were consistent with descriptions of those in Virginia (Mueller 2004), which tend toward “Central Appalachian Rich Cove” or “Red Oak‐Mixed Hardwood”, with some sites showing similarities to goldenseal occurrences in New York (Tait 2006), which tend toward “Sugar maple‐Basswood.” Regionally specific floristic associates identified by ISA appear in part to be related to their overall abundances within the state. Tulip‐poplar dominates on productive mesic sites in the southeastern PA (Burns et al. 1990), while black walnut favors lower slopes and coves underlain by limestone soils (Burns et al. 1990), traits which are consistent with goldenseal sites in the Ridge and Valley province. Black cherry attains its best development on the Allegheny Plateau, where it grows well on all but the very wettest and driest soils (Burns et al. 1990).

Despite slight regional differences in species composition, goldenseal communities were all characterized by high species richness due to their occurrence on deep, mesic, low‐acidity soils (Fike 1999; Zimmerman and Fike 2022). The most prevalent overstory species, tulip‐poplar and sugar maple, are common in rich mesic forests, growing poorly on sites with dry, shallow soils (Burns et al. 1990). The same can be said for many understory associates, including spicebush, rattlesnake fern, black cohosh, wood geranium ( *Geranium maculatum* ), wild ginger ( *Asarum canadense* ), bloodroot, and hairy sweet cicely (Weakley 2023). However, several commonly associated plant taxa could be regarded as “generalists” which occur in a variety of habitats. Associates identified by ISA as significant on soils with “Above Optimum” (> 7) pH levels included those that would be expected only on fertile sites (i.e., tulip‐poplar and rattlesnake fern), as well as species that can commonly be found in acidic sites (i.e., pignut and mockernut hickory). Although plants that reliably grow in fertile soils serve as better indicators of habitat suitability than generalist species, the presence‐only sampling approach used in this study limits the reliability of indicator species individually, and the plant community information presented here should be used collectively, rather than piecemeal, to identify suitable goldenseal habitat for proactive conservation efforts.

The prevalence of non‐native species associated with wild goldenseal populations highlights the widespread shift in plant communities occurring in the northeastern US (Miller et al. 2021). As forest composition transitions from native communities to non‐native species, the utility of historic and current indicator species for site evaluation is likely to be reduced. Furthermore, non‐native plant species have the potential to negatively impact goldenseal performance and overall community floral diversity (Merriam and Feil 2002; Hamelin et al. 2017) particularly in fragmented landscapes in the urbanized southeastern and southwestern portions of the state where goldenseal is more common (Brookings Institution 2003; Pennsylvania Natural Heritage Program 2023). In situ conservation efforts such as assisted migration and forest farming may play an increasingly important role in goldenseal conservation, and the results of this study suggest that the management of non‐native vegetation will concurrently be important. The community analysis results here not only provide a list of potential habitat indicators, but also a baseline for supportive habitat maintenance and restoration efforts.

## Author Contributions

**Ezra Houston:** data curation (equal), formal analysis (equal), investigation (equal), methodology (equal), project administration (equal), validation (equal), visualization (equal), writing – original draft (equal), writing – review and editing (equal). **Eric P. Burkhart:** conceptualization (equal), data curation (equal), funding acquisition (equal), methodology (equal), resources (equal), software (equal), supervision (equal), writing – review and editing (equal). **Xin Chen:** data curation (equal), methodology (equal), software (equal), supervision (equal), validation (equal), writing – review and editing (equal). **Grady Zuiderveen:** conceptualization (equal), data curation (equal), formal analysis (equal), investigation (equal), methodology (equal), writing – original draft (equal), writing – review and editing (equal).

## Conflicts of Interest

The authors declare no conflicts of interest.

## Supporting information


Appendix S1.

## Data Availability

The code used in Maxent modeling, environmental predictor variables, coarse occurrence data, site and soil data, and floristic survey data are available through the link provided here and in Appendix S1: Online Resource 5: https://datadryad.org/stash/share/0TXU6r9E0Zrv2nW_L31dHsKOfVnUoqh_IMfhpfU3MhY. Goldenseal has a special designation of “vulnerable” under 17 Pa. Code § 45.15. Study population coordinates were obtained through a data use agreement with the Pennsylvania Natural Heritage Program, and as such, exact coordinates may not be shared publicly. Provided coordinates have been modified with a random value between −0.1 and 0.1 decimal degrees and rounded to the nearest 0.1 decimal degree.

## Endnotes

^1^

https://www.worldclim.org/data/bioclim.html.

^2^

https://www.nrcs.usda.gov/resources/data‐and‐reports/gridded‐soil‐survey‐geographic‐gssurgo‐database.

^3^

https://www.pasda.psu.edu/uci/DataSummary.aspx?dataset=1247.
