# Supplementary material for: Identification of Goldenseal (Hydrastis canadensis L.) Habitat and Indicators in Pennsylvania, USA: The Influence of Climate and Site on In Situ Conservation of an Edge of Range Plant Species
Source: Ecol Evol. 2025 Feb 28;15(3):e71050. doi: 10.1002/ece3.71050 (PMC11868700; doi:10.1002/ece3.71050)
Supplement: Supplementary file 1 — Appendix S1. [file ECE3-15-e71050-s001.pdf]

Title: Identification of goldenseal (*Hydrastis canadensis* L.) habitat and indicators in Pennsylvania, U.S.A: the influence of climate and site on *in situ* conservation of an edge of range plant species.

Journal Name: Ecology and Evolution

Online Resource 1. List of all variables used in initial Maxent model run.

Variable	Description/Source
	Climatic Predictors
BIO1	Annual Mean temperature (°C)
BIO2	Mean Diurnal Range (Mean of monthly (max temp - min temp))
BIO3	Isothermality (BIO2/BIO7) (* 100)
BIO4	Temperature Seasonality (standard deviation *100)
BIO5	Max Temperature of Warmest Month
BIO6	Min Temperature of Coldest Month
BIO7	Temperature Annual Range (BIO5-BIO6)
BIO8	Mean Temperature of Wettest Quarter
BIO9	Mean Temperature of Driest Quarter
BIO10	Mean Temperature of Warmest Quarter
BIO11	Mean Temperature of Coldest Quarter
BIO12	Annual Precipitation
BIO13	Precipitation of Wettest Month
BIO14	Precipitation of Driest Month
BIO15	Precipitation Seasonality (Coefficient of Variation)
BIO16	Precipitation of Wettest Quarter
BIO17	Precipitation of Driest Quarter
BIO18	Precipitation of Warmest Quarter
BIO19	Precipitation of Coldest Quarter
	Edaphic Predictors
Band_1.1	Available water capacity (cm)
Band_1.2	Bedrock type
Band_1.3	Soil bulk density (g/cm^3)
Band_1.4	Percent clay
Band_1.5	Depth to bedrock
Band_1.6	Soil erodability factor, rock fragment free
Band_1.7	Length of the frost free period
Band_1.8	Organic matter content (% by weight)
Band_1.9	Soil permeability rate (cm/hr)
Band_1.10	Soil pH
Band_1.11	Percent sand
Band_1.12	Percent soil passing sieve no.10 (coarse)
Band_1.13	Percent soil passing sieve no.200 (fine)
Band_1.14	Percent silt
Band_1.15	Soil slope(%) of a soil component
Band_1.16	Soil order based on USDA soil taxonomy
Band_1.17	Soil suborder based on USDA soil taxonomy

	Topographic Predictors
Band_1.1.1	Transformed aspect according to Beer et al. (1966)
Band_1.2.1	Elevation relief ratio (15-pixel neighborhood)
Band_1.3.1	Integrated soil moisture index (Iverson et al. 1997)
Band_1.4.1, 1.5.1	Variance of elevation (15-pixel, 27-pixel neighborhood)
Band_1.6.1	Slope x COS(Aspect)
Band_1.7.1	Slope x SIN(Aspect)
Band_1.8.1, 1.9.1, 1.10.1, 1.11.1, 1.12.1	Topographic position indices at scales of 150m, 300m, 500m, 1000m, and 2000m
Band_1.13.1	Topographic radiation index

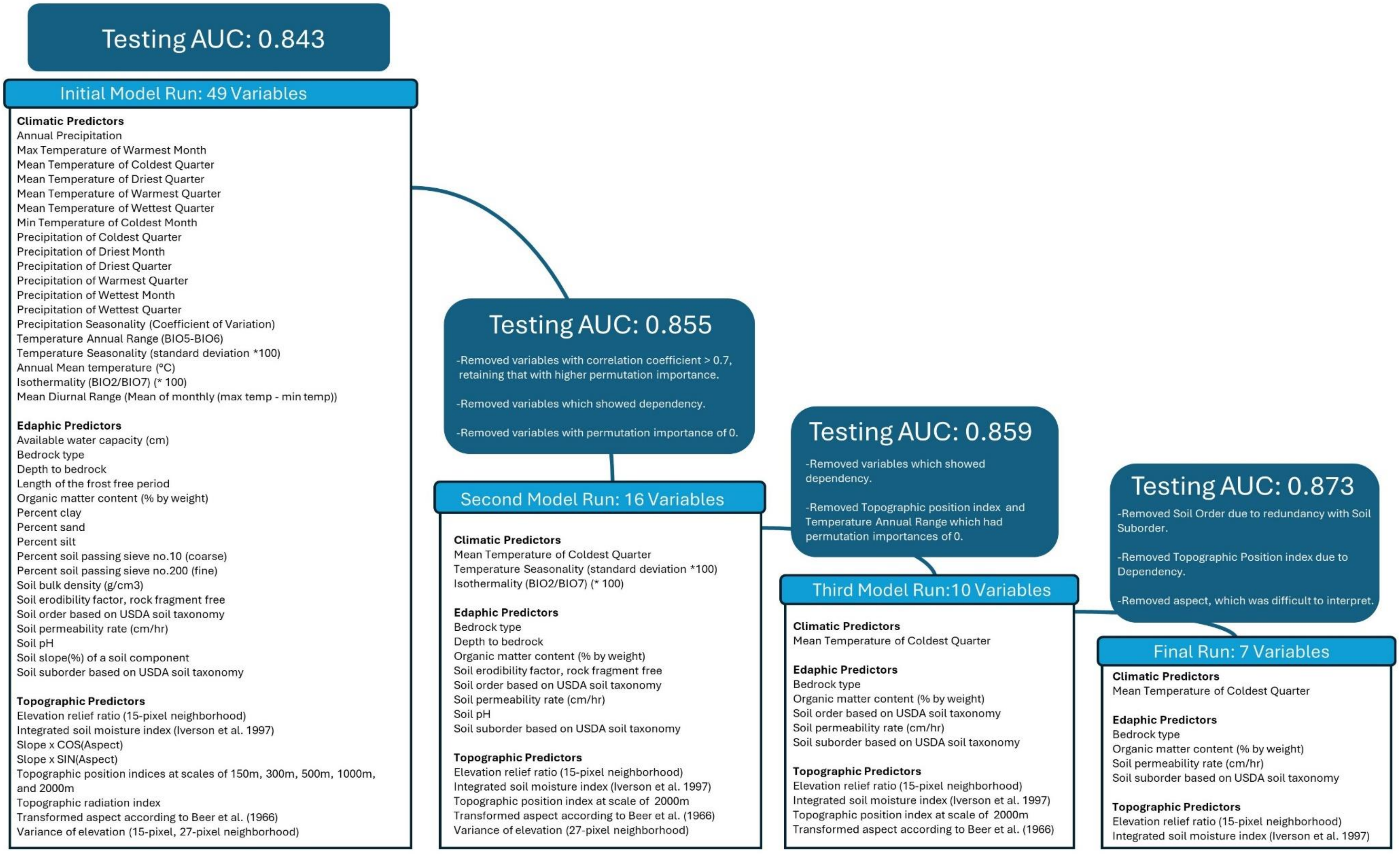

Online Resource 2: Variable selection process for the goldenseal suitability model in Maxent.

Online Resource 3. Study sites, population traits and habitat characteristics associated with goldenseal in Pennsylvania.

Site Name	Population Size and Area	Number of sample plots	Site Description	Aspect	Elevation (M)	Topographic position
Allegheny-SH	500 ramets, 20% reproductive, <1 hectare	3	Moist lower-slope	W, SW	251	lower-slope
Beaver-CM	1,000 ramets, 20% reproductive, >1 hectare	2	Moist side-slope bench	N, NW	235	upper-slope
Bedford-BM	5,000 ramets, 5% reproductive, >1 hectare	3	Mid-slope, recent logging	W	423	mid-slope
Bedford-WW	10,000 ramets, 15% reproductive, >1 hectare	4	Moist forest farmed site	none	348	flat
Bucks-QT	5,000-10,000 ramets, 10% reproductive, >1 hectare	4	Moist lowland	none	119	flat
Butler-JE	100-500 ramets, 30% reproductive, <1 hectare	1	Moist lower-slope	W	341	lower-slope
Cambria-GL	100-500 ramets, 5% reproductive, <1 hectare	1	Moist ridgetop	none	733	ridgetop
Cambria-JK	500-1,000 ramets, 25% reproductive <1 hectare	1	Upland forest farmed site	none	521	top of a hill
Centre-ST	>10,000 ramets, 5% reproductive, >1 hectare	4	Moist side-slope on drainage bench	NW	336	mid-slope
Chester-LF	>10,000 ramets, 5% reproductive, >1 hectare	4	Moist lower-slope to dry ridge	N, NE	104	lower, mid, and upper-slope

Clarion-LH	50-100 ramets, <5% reproductive, <1 hectare	1	Mid-slope in steep ravine	E	319	mid-slope
Dauphin-JM	50-100 ramets, 60% reproductive, <1 hectare	1	Moist lowland	none	213	flat
Huntingdon-MG	5,000 ramets, 15% reproductive, >1 hectare	4	Gently sloping mountain hollow	none	232	hollow bottom
Indiana-PR	5,000-10,000 ramets, <5% reproductive, >1 hectare	4	Moist toe-slope, lowland	N	357	lower-slope, valley bottom
Lancaster-BW	1,000 ramets, 15% reproductive, <1 hectare	4	Moist lowland	none	115	flat
Lancaster-MV	1,000-5,000 ramets, 15% reproductivem <1 hectare	1	Moist lowland	none	176	flat
Lebanon-GD	10-50 ramets, <5% reproductive, <1 hectare	1	Moist flat site	none	201	flat
Lebanon-GL	100 ramets, 5% reproductive, <1 hectare	1	Wet lowland	none	188	flat
Mifflin-JB	1,000 ramets, 10% reproductive, <1 hectare	3	Moist sinkhole	SE	196	lower-slope
Montgomery-BL	1,000-5,000 ramets, <5% reproductive, 1 hectare	1	Moist lower-slope	N	40	lower-slope
Montgomery-CD	100 ramets, <5% reproductive, <1 hectare	1	Moist lowland	none	57	flat

Montgomery-GL	100-500 ramets, 15% reproductive, 1 hectare	1	Moist mid-slope	NE	58	mid-slope
Montgomery-SX	100-500 ramets, 10% reproductive, <1 hectare	1	Moist mid-slope bench	NW	33	mid-slope
Westmoreland-DP	100-500 ramets, 10% reproductive, <1 hectare	1	Moist mid-slope	N	329	mid-slope
Westmoreland-MA	>10,000 ramets, 5% reproductive, 1 hectare	2	Moist flat site	none	287	flat
Westmoreland-PM	500-1,000 ramets, 15% reproductive, <1 hectare	1	Wet lowland	none	377	flat
York-AB	1,000 ramets, 5% reproductive, 1 hectare	1	Wet lowland	none	183	flat
York-GP	5,000 ramets, 5% reproductive, >1 hectare	3	Dry side-slope and moist bench	SE, NW	133	mid-slope

Online Resource 4. Frequency of species identified as being associated with goldenseal in Pennsylvania (n = 58 plots)**.**

**Scientific name**[+]	**Common name**	**# of plots**
Lindera benzoin (L.) Blume	Spicebush	44
Parthenocissus quinquefolia (L.) Planch	Virginia Creeper	43
Arisaema triphyllum (L.) Schott	Jack-in-the-pulpit	42
Berberis thunbergii DC.*	Japanese barberry	31
Podophyllum peltatum L.	Mayapple	31
Botrypus virginianus (L.) Michx.	Rattlesnake fern	29
Dryopteris marginalis (L.) A. Gray	Marginal wood fern	29
Amphicarpa bracteata (L.) Fernald	American hogpeanut	27
Rosa multiflora Thunb. ex Murray *	Multiflora rose	27
Toxicodendron radicans (L.) Kuntze	Poison ivy	27
Actaea racemosa L.	Black cohosh	25
Microstegium vimineum (Trin.) A. Camus *	Japanese stiltgrass	25
Polystichum acrostichoides (Michx.) Schott	Christmas fern	24
Solidago/Symphiotrichum spp.	Goldenrod or aster spp.	24
Uvularia perfoliata L.	Perfoliate bellwort	24
Galium circaezens Michx.	Licorice bedstraw	23
Liriodendron tulipifera L.	Tulip-poplar	23
Viola pubescens Ait.	Downy yellow violet	23
Acer saccharum Marshall	Sugar maple	22
Geranium maculatum L.	Wood geranium	22
Viola hirsutula Brainerd	Southern woodland violet	22
Alliaria petiolata (M. Bieb.) Cavara & Grande *	Garlic mustard	21
Circaea canadensis L.	Enchanter's nightshade	21
Sanicula spp. L.	Black snakeroot	21
Asarum canadense L.	Wild ginger	19
Persicaria longiseta (Bruijn) Kitagawa *	Bristled knotweed	19
Sanguinara canadensis L.	Bloodroot	19
Maianthemum racemosum (L.) Link	False Solomon's seal	18
Agrimonia spp. Tourn. ex L.	Agrimony	17
Nabalus spp. Cass.	Rattlesnake root	17
Osmorhiza claytonii (Michx.) C.B. Clarke	Hairy sweet cicely	17
Persicaria virginiana (L.) Gaertn.	Virginia jumpseed	15
Uvularia sessilifolia L.	Sessile bellwort	15
Geum canadense Jacq.	White avens	14
Hepatica americana (DC.) Ker Gawl.	Round-lobed hepatica	14
Osmorhiza longistylis (Torr.) DC.	Wild anise	14
Phryma leptostachya L.	Lopseed	14
Thalictrum thalictroides (L.) A.J. Earnes & B. Boivin	Rue-anemone	14

Dioscorea villosa L.	Wild yam	13
Eurybia divaricata (L.) G.L. Nesom	White wood aster	13
Galium triflorum Michx.	Sweet bedstraw	13
Juglans nigra L.	Black walnut	13
Impatiens sp. (I. pallida or *I. capensis*) L.	Jewelweed species	12
Collinsonia canadensis L.	Stoneroot	11
Oxalis acetosella L.	Wood sorrel	11
Pilea pumila (L.) A. Gray	Clearweed	11
Galearis spectabilis (L.) Raf.	Showy orchid	10
Ageratina altissima (L.) King & H.E. Robins.	White snakeroot	9
Cardamine concatenata (Michx.) O. Schwarz	Cutleaf toothwort	9
Carya cordiformis (Wang) K. Koch	Bitternut hickory	9
Quercus alba L.	White oak	9
Quercus rubra L.	Northern red oak	9
Carya glabra (Mill.) Sweet	Pignut hickory	8
Erythronium americanum Ker-Gawl.	Yellow trout-lily	8
Hackelia virginiana I.M. Johnston	Beggar's lice	8
Impatiens capensis Meerb.	Common jewelweed	8
Viburnum acerifolium L.	Maple-leaf viburnum	8
Adiantum pedatum L.	Maidenhair fern	7
Claytonia virginica L.	Virginia springbeauty	7
Monarda didyma L.	Scarlet beebalm	7
Monotropa uniflora L.	Ghost pipe	7
Panax quinquefolius L.	American ginseng	7
Prosartes lanuginosa (Michx.) D. Don	Yellow mandarin	7
Prunus serotina L.	Black cherry	7
Smilax hispida Raf.	Bristly greenbriar	7
Vitis spp. L.	Wild grape	7
Cryptotaenia canadensis (L.) DC.	Canadian honewort	6
Lonicera japonica Thunb. *	Japanese honeysuckle	6
Polygonatum biflorum (Walt.) Ell.	Smooth Solomon's-seal	6
Smilax herbacea L.	Smooth carrionflower	6
Tilia americana L.	American basswood	6
Allium tricoccum Aiton	Ramps	5
Carya ovata (Mill.) K. Koch	Shagbark hickory	5
Carya tomentosa (Poir.) Nutt.	Mockernut hickory	5
Fragaria vesca L. *	Wild strawberry	5
Maianthemum canadense Desf.	Canada mayflower	5
Polygonatum pubescens (Willd.) Pursh	Hairy Solomon's-seal	5
Polygonatum sp. (P. biflorum or *P. pubescens)* Mill.	Solomon's-seal species.	5
Trillium erectum L.	Purple trillium	5
Ulmus rubra Muhl.	Slippery elm	5

Amauropelta noveboracensis (L.) S.E. Fawc. & A.R. Sm.	New York fern	4
Celastrus orbiculatus Thunb. *	Oriental bittersweet	4
Desmodium spp. Desv.	Tick trefoil	4
Mitchella repens L.	Partridge berry	4
Onoclea sensibilis L.	Sensitive fern	4
Rubus phoenicolasius Maxim. *	Wineberry	4
Sedum ternatum Michx.	Woodland stonecrop	4
Smilax rotundifolia L.	Greenbriar	4
Acer platanoides L. *	Norway maple	3
Actaea pachypoda Elliott	White baneberry	3
Anemone quinquefolia L.	Wood anemone	3
Caulophyllum thalictroides L.	Blue cohosh	3
Clinopodium vulgare L.	Wild basil	3
Euonymus alatus (Thunb.) Siebold *	Winged euonymous	3
Fagus grandifolia Ehrhart	American beech	3
Fraxinus pennsylvanica Marshall	Green ash	3
Laportea canadensis (L.) Wedd.	Wood nettle	3
Phegopteris hexagonoptera (Michx.) Fée	Beech fern	3
Quercus montana Willd.	Chestnut oak	3
Solidago flexicaulis L.	Broadleaved goldenrod	3
Trillium grandiflorum (Michx.) Salisb.	Large-flowered trillium	3
Tussilago farfara L. *	Coltsfoot	3
Aralia racemosa L.	American spikenard	2
Artemisia sp. L. *	Mugwort	2
Erechtites hieracifolius (L.) Raf. ex DC.	Pilewort	2
Eutrochium spp. Raf.	Joe-pye weed	2
Fraxinus americana L.	White ash	2
Fraxinus nigra Marshall	Black ash	2
Hamamelis virginiana L.	Witch-hazel	2
Helianthus spp. L.	Sunflower	2
Magnolia acuminata L.	Cucumber tree	2
Packera aurea (L.) W.A. Weber & Á. Löve	Golden ragwort	2
Pinus strobus L.	Eastern white pine	2
Ranunculus abortivus L.	Littleleaf buttercup	2
Sassafras albidum (Nutt.) Nees	Sassafras	2
Tiarella cordifolia L.	Heartleaf foamflower	2
Viburnum prunifolium L.	Blackhaw	2
Viola rostrata Pursh	Longspur violet	2
Viola striata Aiton	Striped violet	2
Acer pensylvanicum L.	Striped maple	1
Anemone canadensis L.	Canada anemone	1
Anemone virginiana L.	Tall thimbleweed	1

Aplectrum hyemale (Muhl. ex Willd.) Torr.	Putty-root orchid	1
Blephilia ciliata (L.) Benth.	Downy wood mint	1
Campanula americana L.	Tall bellflower	1
Clintonia umbellulata (Michx.) Morong	White clintonia	1
Crataegus sp. L.	Hawthorn	1
Cynoglossum virginianum L.	Wild comfrey	1
Daucus carota L. *	Wild carrot	1
Desmodium nudiflorum (L.) DC.	Naked-flowered tick trefoil	1
Dicentra cucullaria (L.) Bernh.	Dutchman's breeches	1
Eupatorium altissimum L.	Tall boneset	1
Fallopia sp. Adans.	Bindweed species	1
Galium aparine L.	Sticky willy	1
Galium lanceolatum (Torr. & A. Gray) Torr.	Lanceleaf wild licorice	1
Hesperis matronalis L. *	Dame's rocket	1
Hydrophyllum virginianum L.	Virginia waterleaf	1
Impatiens pallida Nutt.	Yellow jewelweed	1
Lysimachia ciliata L.	Fringed loosestrife	1
Medeola virginiana L.	Indian cucumber-root	1
Menispermum canadense L.	Common moonseed	1
Obolaria virginica L.	Virginia pennywort	1
Persicaria perfoliata (L.) H. Gross *	Mile-a-minute weed	1
Phlox divaricata L.	Woodland phlox	1
Phytolacca americana L.	American pokeweed	1
Picea abies (L.) H. Karst. *	Norway spruce	1
Plantago sp. L.	Plantain	1
Prunella vulgaris L.	Self-heal	1
Prunus avium L. *	Bird cherry	1
Robinia pseudoacacia L.	Black locust	1
Rubus occidentalis L.	Black raspberry	1
Staphylea trifolia L.	American bladdernut	1
Symplocarpus foetidus Salisb.	Skunk cabbage	1
Teucrium canadense L.	American germander	1
Thalictrum dioicum L.	Early meadow-rue	1
Tsuga canadensis (L.) Carrière	Eastern hemlock	1
Verbena urtisifolia L.	Nettle-leaved vervain	1
Viola blanda Willd.	Sweet white violet	1
Viola canadensis L.	Canadian white violet	1

Asterisk (*) denote non-native, exotic species.

+All taxonomy follows Weakley, 2023.

Online Resource 5. Open access data: https://doi.org/10.5061/dryad.9s4mw6mrb
